# Supplementary material for: Effects of network topology on the performance of consensus and distributed learning of SVMs using ADMM
Source: PeerJ Comput Sci. 2021 Mar 9;7:e397. doi: 10.7717/peerj-cs.397 (PMC7959654; doi:10.7717/peerj-cs.397)
Supplement: Supplemental Information 1 [file peerj-cs-07-397-s001.pdf]

SUPPLEMENT

Performance of the algorithm for Regular and Complete Graphs

Results for `cod-rna`

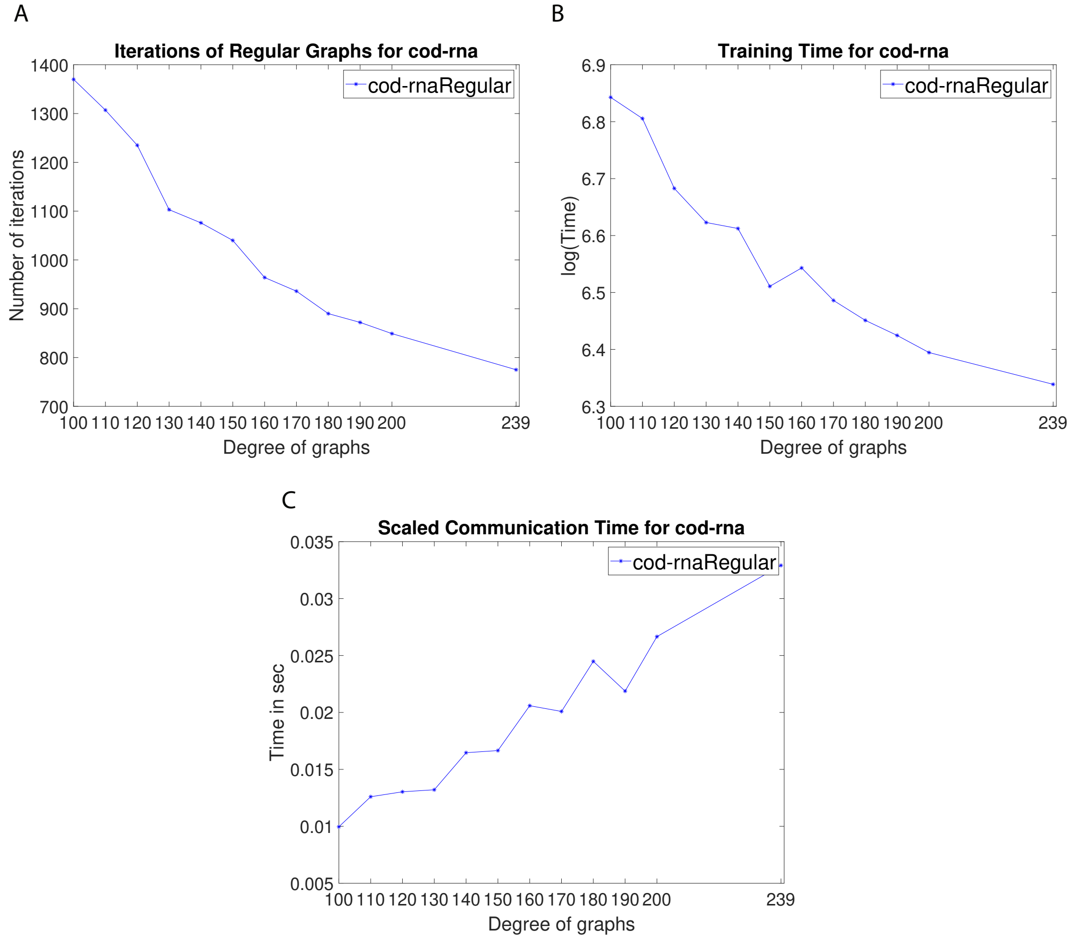

Figure 1. Iterations, total training and scaled communication time for `Cod-rna` using regular graphs. (A) Iterations, (B) Training time, and (C) Scaled communication time during training.

Results for Seismic

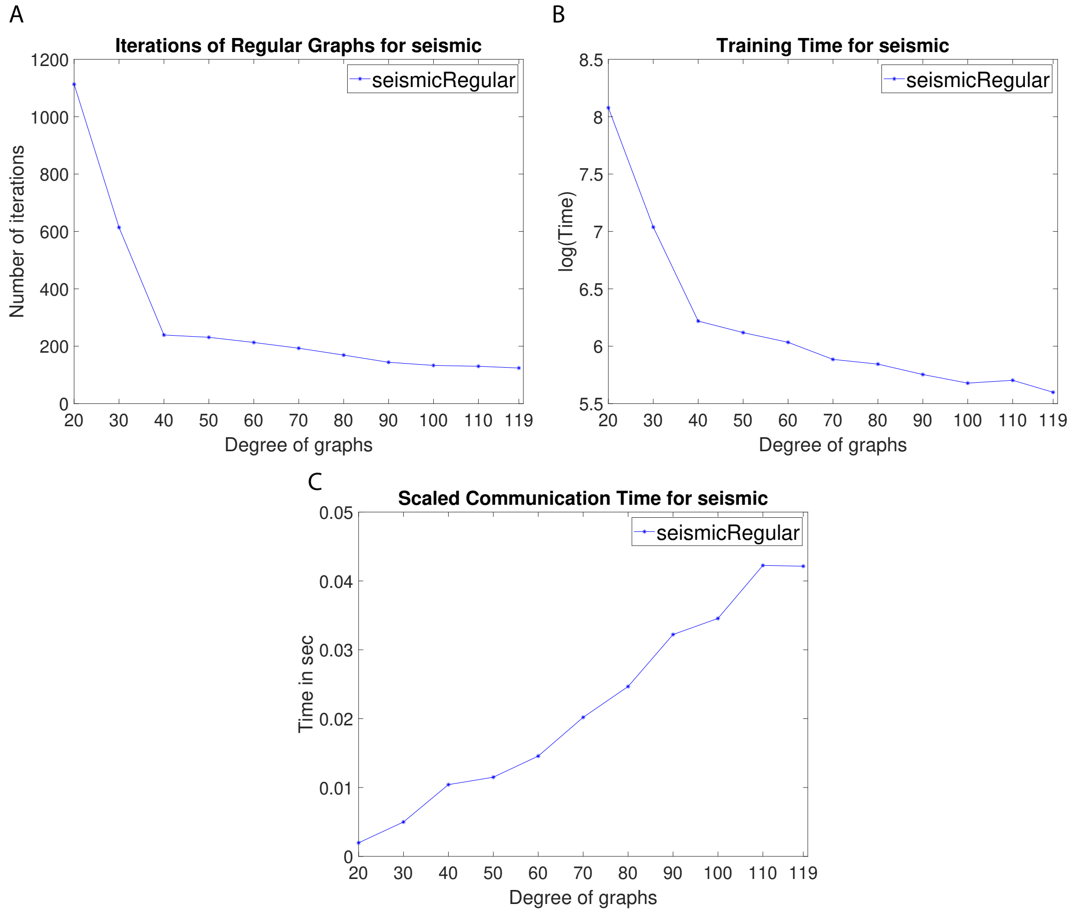

Figure 2. Iterations, total training and scaled communication time for `Seismic` using regular graphs. (A) Iterations, (B) Training time, and (C) Scaled communication time during training.

Results for `pre-miRNAs`

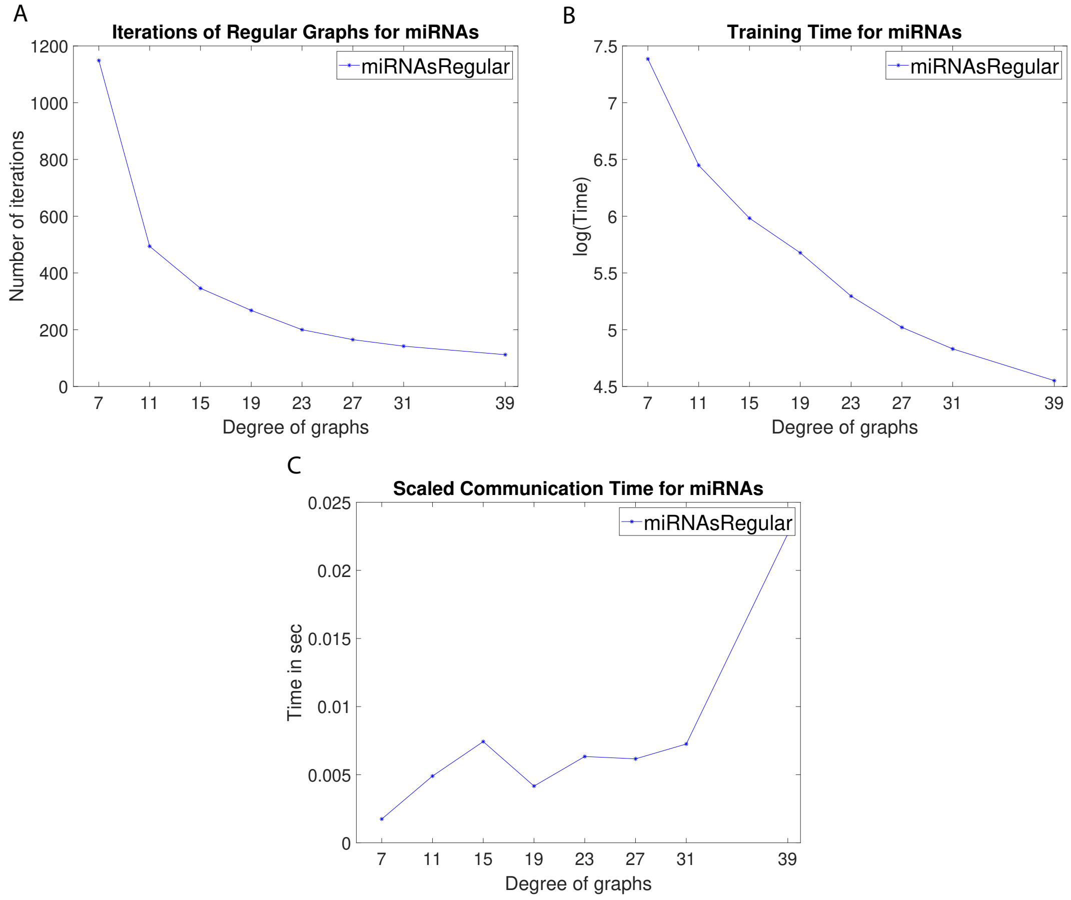

Figure 3. Iterations, total training and scaled communication time for `pre-miRNAs` using regular graphs. (A) Iterations, (B) Training time, and (C) Scaled communication time during training.

Number of iteration and scaled communication time for higher degree graphs

Results for `Susy` and `Cod-rna`

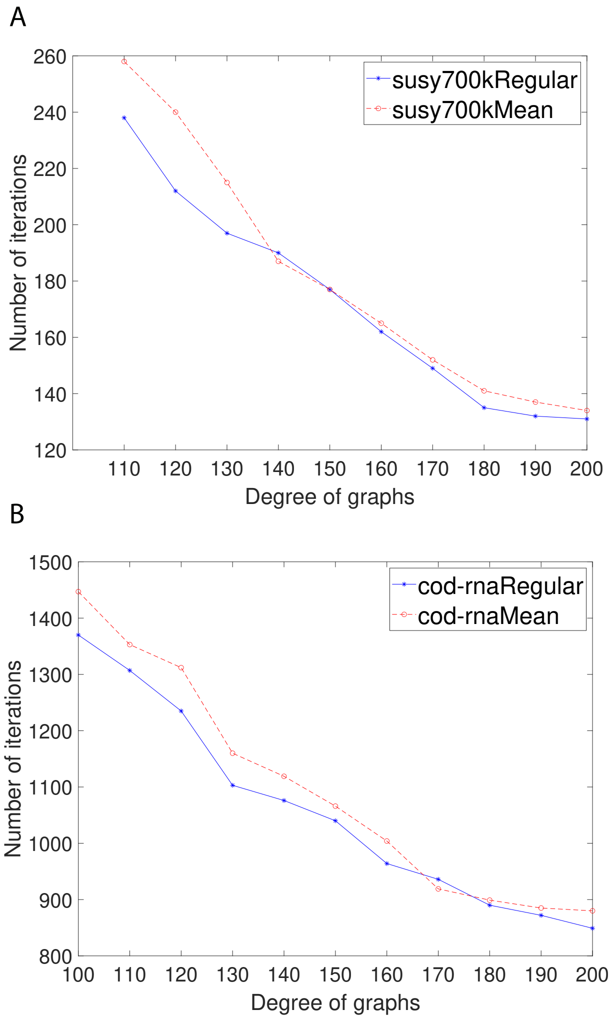

Figure 4. Iterations for regular and mean degree graphs for `Susy` and `Cod-rna` datasets using 240 graph nodes. (A) Number of iteration for different degrees for `Susy` and (B) Number of iteration for different degrees for `Cod-rna`.

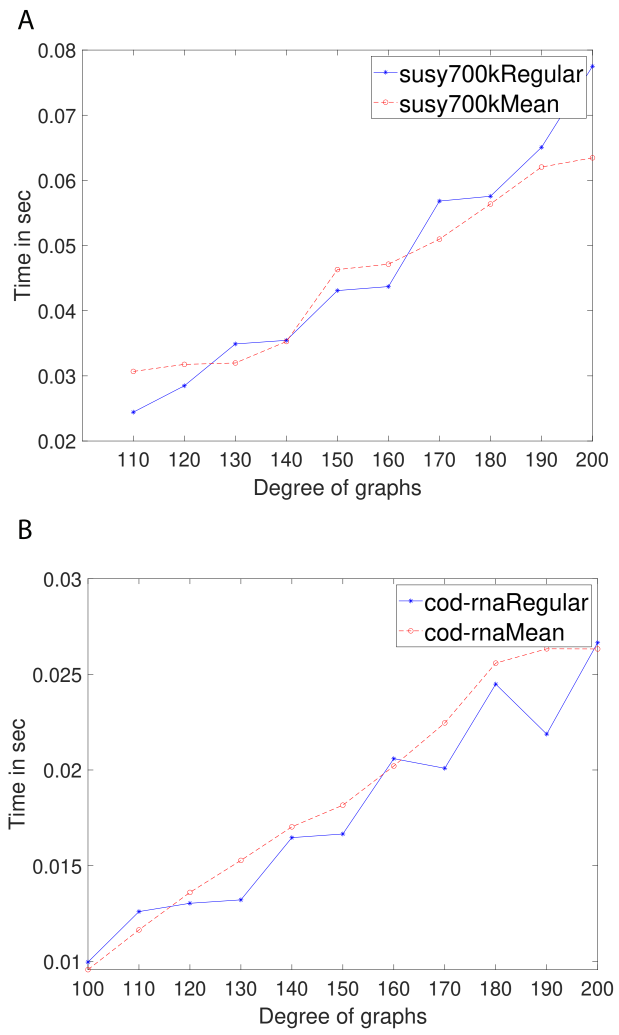

Figure 5. Scaled communication time for different degrees for `Susy` and `Cod-rna` using 240 graph nodes. (A) Scaled communication time for `Susy` and (B) Scaled communication time for `Cod-rna`.

DIFFERENCE BETWEEN THE RESULTS OF EACH NODE VJ AND THE AVERAGE RESULTS OF THE NEIGHBOURING NODES V

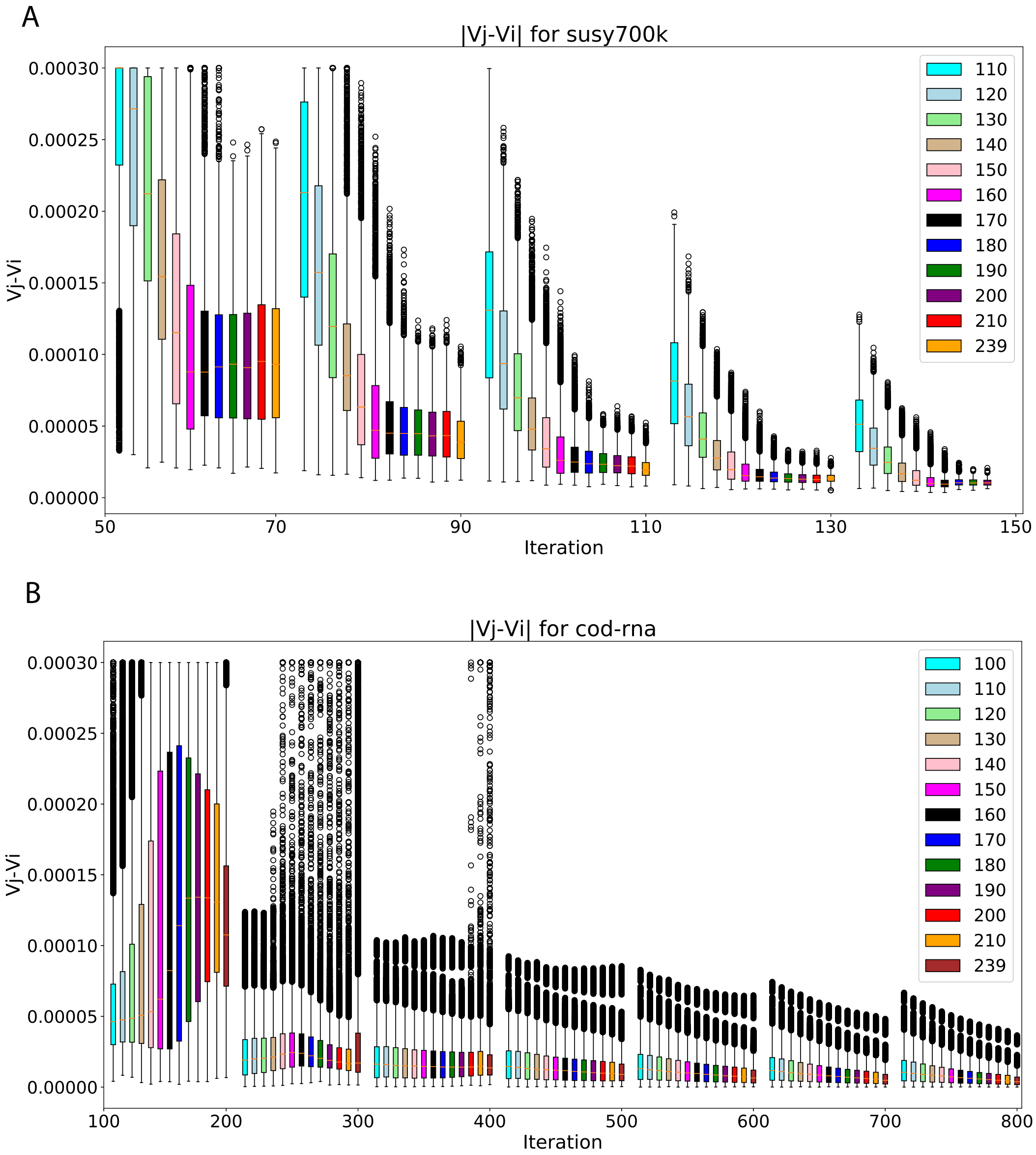

Figure 6. The difference between the result of node j, V_j, with the average result of its neighboring nodes, V_i, i.e., $|V_j - V_i|$, per iteration for Susy and Cod-rna datasets using regular graphs and 240 graph nodes. (A) $|V_j - V_i|$ per iteration for Susy and (B) $|V_j - V_i|$ per iteration for Cod-rna.

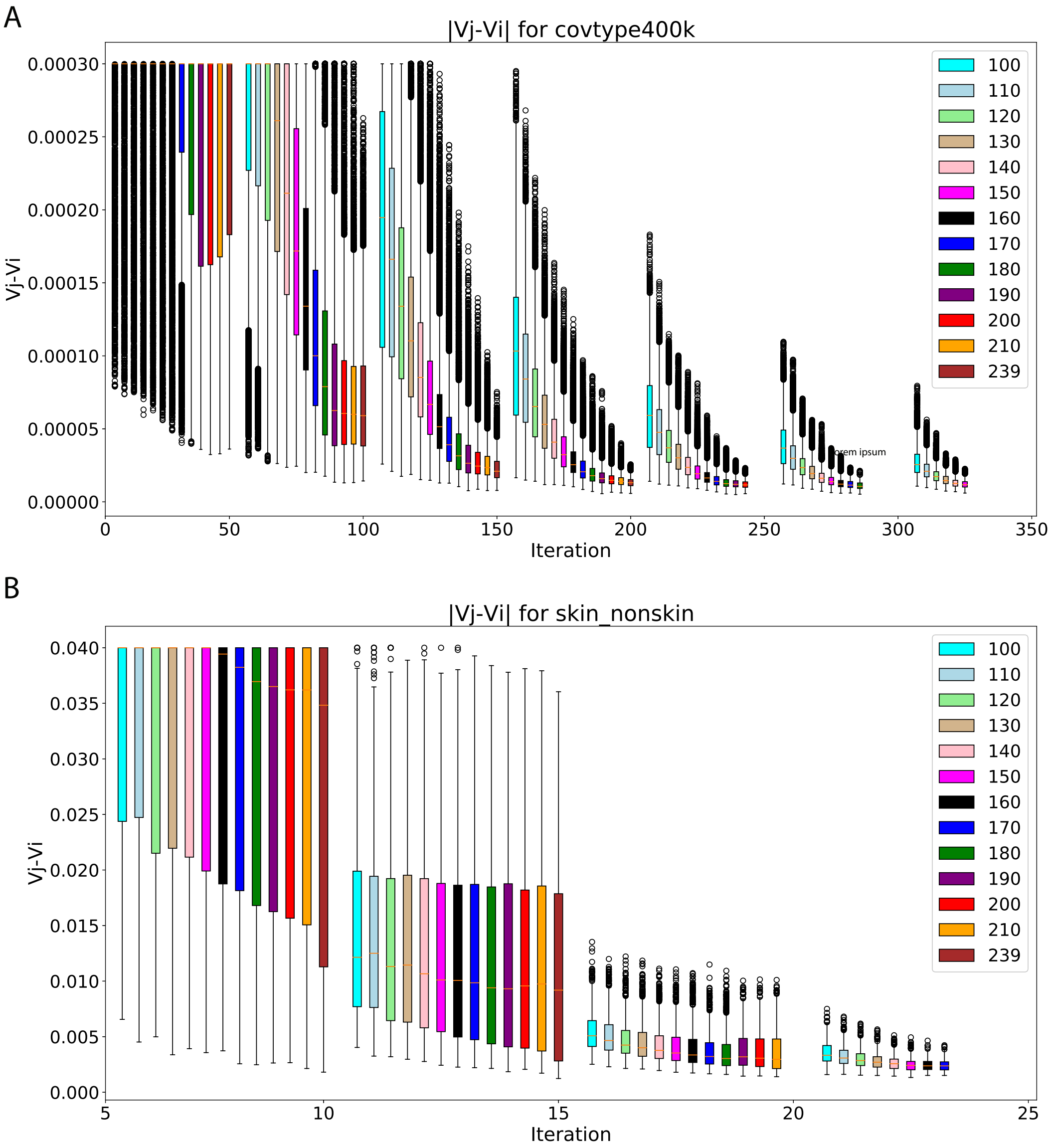

Figure 7. The difference between the result of node j, V_j, with the average result of its neighboring nodes, V_i, i.e., $|V_j - V_i|$, per iteration for `Covtype` and `SkinNonskin` datasets using regular graphs and 240 graph nodes. (A) $|V_j - V_i|$ per iteration for `Covtype` and (B) $|V_j - V_i|$ per iteration for `SkinNonskin`.
